# Cis-existence of H3K27me3 and H3K36me2 in mouse embryonic stem cells revealed by specific ions of isobaric modification chromatogram

**DOI:** 10.1186/s13287-015-0131-0

**Published:** 2015-07-21

**Authors:** Hailei Mao, Gang Han, Longyong Xu, Duming Zhu, Hanqing Lin, Xiongwen Cao, Yi Yu, Charlie Degui Chen

**Affiliations:** Department of Anesthesiology and Critical Care Medicine, Zhongshan Hospital, Fudan University, 180 Fenglin Road, Shanghai, 200032 China; State Key Laboratory of Molecular Biology, and Shanghai Key laboratory of Molecular Andrology, Institute of Biochemistry and Cell Biology, Shanghai Institutes for Biological Sciences, Chinese Academy of Sciences, 320 Yueyang Road, Shanghai, 200031 China

## Abstract

**Introduction:**

Histone H3 lysine 27 trimethylation (H3K27me3) and H3 lysine 36 trimethylation (H3K36me3) are important epigenetic modifications correlated with transcription repression and activation, respectively. These two opposing modifications rarely co-exist in the same H3 polypeptide. However, a small but significant amount of H3 tails are modified with 5 methyl groups on K27 and K36 in mouse embryonic stem cells (mESCs) and it is unclear how the trimethylation is distributed on K27 or K36.

**Methods:**

A label-free, bottom-up mass spectrum method, named specific ions of isobaric modification chromatogram (SIMC), was established to quantify the relative abundance of K27me2-K36me3 and K27me3-K36me2 in the same histone H3 tail.

**Results:**

By using this method, we demonstrated that the H3K27me3-K36me2 comprises about 85 % of the penta-methylated H3 tails at K27 and K36 in mESCs. Upon mESC differentiation, the abundance of H3K27me3-K36me2 significantly decreased, while the level of H3K27me2-K36me3 remains unchanged.

**Conclusion:**

Our study not only revealed the cis-existence of H3K27me3-K36me2 in mESCs, but also suggested that this combinatorial histone modification may assume a specific regulatory function during differentiation.

**Electronic supplementary material:**

The online version of this article (doi:10.1186/s13287-015-0131-0) contains supplementary material, which is available to authorized users.

## Introduction

Histone methylation is a complex modification that regulates transcription and chromatin dynamics [1–3]. Methylation can occur at lysine and arginine residues in histone proteins. Each lysine residue can have three states of methylation, having one (mono), two (di), or three (tri) methyl groups covalently attached to the amine group of the lysine side chain, and the arginine residue can be monomethylated, or symmetrically or asymmetrically dimethylated [4–6]. Depending on the specific residues and modification states, histone methylation can repress or activate transcription [2, 7]. For example, histone H3 lysine 27 trimethylation (H3K27me3) is associated with transcriptional repression, whereas histone H3 lysine 36 trimethylation (H3K36me3) is associated with transcriptional activation [3, 8, 9].

Genome-wide chromatin immunoprecipitation sequencing (ChIP-Seq) experiments revealed that higher methylation states of H3K27 (H3K27me2/3) and H3K36 (H3K36me2/3) rarely colocalized at the same genomic locus, consistent with their opposing functions [8, 9]. However, it was recently reported that two polycomb-like proteins, PHF1 and PHF19, can target polycomb repressive complex 2 (PRC2) to catalyze H3K27me3 through binding to H3K36me3 in a subset of developmental genes in embryonic stem cells (ESCs) [10], indicating an important regulatory interplay between these two opposing marks. In fact, H3K27me2/3 and H3K36me2 are found colocalized in the same nucleosome in ESCs, mouse embryonic fibroblast (MEF) cells, and HeLa cells [11]. These results suggest that the higher methylation states at H3K27 and H3K36 residues can form a combinatorial modification cis-existing at the same H3 and may also play a crucial role in the embryonic development. Although histone H3 peptide containing both H3K27 and H3K36 sites with five methyl groups was also detected by mass spectrometry (MS) [11, 12], the assignment of the five methyl groups in H3K27 and H3K36 in ESCs and during differentiation is currently unknown.

Quantification of histone methylations can be achieved by a bottom-up MS strategy [13, 14]. In this approach, individual or intact histones are digested by site-specific proteases to generate a peptide containing a modified residue of interest and the amount of peptide is quantified at the MS level. However, the current digestion methods cannot always generate a peptide with only one modification, because histones are densely modified on their short N-terminal tails [15, 16]. For example, trypsin or Arg-C digestion can produce a peptide from H3K27 to H3R40 (K27-R40) that contains K27 and K36, both of which are subject to methylation. If H3K27-R40 fragments contain five methyl groups (me5), there are two possible patterns: K27me2-K36me3 and K27me3-K36me2. Although the level of H3K27-R40 with a certain number of methyl groups can be quantified by selected ion chromatogram (SIC) at the MS level, whether the methyls are on K27 or K36 cannot be determined. Fragments with the same number of modified groups but different patterns of distribution are called isobarically modified peptides [14, 15].

Quantification of isobarically methylated H3K27-R40 peptides has been attempted by increasing the elution time in reversed phase high-performance liquid chromatography (RP-HPLC) [15]. Using this method, several isobarically modified peptides were artificially divided into different fractions by liquid chromatography (LC) and quantified. Since each isoform of the isobarically modified peptides behaves virtually identically in RP-HPLC [14], the assignment of the isoform is subjective and therefore the quantification is unreliable [15]. Another method to quantify isobarically methylated H3K27-R40 peptides is by propionic anhydride labeling [11, 12, 14]. Propionic anhydride is used to add propionyl group to the N-terminal, unmodified and monomethylated lysines, and then trypsin is used to produce the target H3K27-R40 peptides. For some isobarically modified peptides, the propionylated isoforms will have different RP-HPLC retention times and masses, which can be subjected to separation and quantification by SIC at the MS level. However, this technology cannot quantify all forms of isobarically modified peptides; for example, H3K27me2-K36me3 and H3K27me3-K36me2 cannot be distinguished [11, 12, 14]. Effective methods for systemic quantification of the isobarically modified peptides are therefore still lacking.

In this study, we developed a label-free, bottom-up MS method that can quantify the relative abundance of K27me2-K36me3 and K27me3-K36me2 in the same histone H3 tail. Using this method, the specific ions of isobaric modification chromatogram (SIMC), H3K27me3-K36me2 was shown to be the major form, comprising about 85 % of pentamethylated H3 tails at K27 and K36 sites in mouse embryonic stem cells (mESCs). Moreover, the abundance of H3K27me3-K36me2 significantly decreased upon mESC differentiation, suggesting a specific regulatory function in the process.

## Methods

### LC-MS/MS for synthetic isobarically methylated histone peptides

Two synthetic isobarically methylated peptides of K_27_SAPATGGVKKPHR_40_me5 (K27-R40me5) from histone H3.1 or H3.2 with a purity of more than 90 %, S1 (K27me2-K36me3) and S2 (K27me3-K36me2), were diluted to the same concentration. The two peptides were then mixed in weight ratios of 3:1 (M1), 1:1 (M2), 1:3 (M3), and 1:6 (M4). The individual peptide and their mixtures including 75 ng H3K27-R40me5 peptides were analyzed by LC-tandem mass spectrometry (MS/MS) using a Waters nanoAcquity UPLC system (Waters, Milford, Massachusetts, USA) interfaced to a LTQ-Orbitrap instrument (ThermoFisher Scientific, Bremen, Germany) and run in a positive ion mode. The nanoliter flow LC was operated in a CapTrap (MICHROM, Bioresources, Inc., Auburn, California, USA) and one following analytical column (75 μm × 15 cm) packed with C18 reverse-phase material (Ultimate C18, 100 Å, 3 μm spherical particles). Solvent A was double-distilled water (ddH_2_O) with 0.1 % formic acid, and solvent B was acetonitrile (ThermoFisher Scientific, Bremen, Germany) with 0.1 % formic acid. Samples were injected into solvent A at a flow rate of 300 nl/minute. Peptides were separated with a gradient of 5–35 % solvent B from 0–25 minutes. The full-scan MS spectra were acquired in Orbitrap with a resolution of 60,000 for mass-to-charge ratio (*m/z*) and 400 full width at half maximum (FWHM). The full MS mass range was 300–1600, AGC target 1,000,000, and maximum injection time 500 milliseconds. The three most intense ions with a parent *m/z* value of K27-R40me5 carrying charge 3+ (501.9755) were isolated for fragmentation in the Orbitrap with a resolution of 15,000 (high resolution) and LTQ (low resolution) using collision-induced dissociation (CID; with a normalized collision energy of 35 %, and an isolation window of 2.00 units), respectively. The dynamic exclusion time was not enabled.

### LC-MS/MS for tryptic synthetic isobarically methylated histone peptides

Two synthetic isobarically methylated peptides of L_20_ATKAARKSAPATGGVKKPHRYRPGTVALREIR_52_me5 (L20-R52me5) from histone H3.1 or H3.2 with a purity of more than 90 %, L1 (K27me2-K36me3) and L2 (K27me3-K36me2), were diluted to the same concentration. The two long peptides were mixed in weight ratios of 3:1, 1:1, 1:3, and 1:6, and samples with a total of 2.5 μg mixture were digested with trypsin (V511C; Promega, Madison, Wisconsin, USA) at a substrate/enzyme ratio of 200:1 at 37 °C for 20, 40, and 120 minutes, respectively. The tryptic samples including 150 ng H3L20-R52me5 peptides were analyzed by LC-MS/MS using the same LTQ-Orbitrap instrument (ThermoFisher Scientific), LC gradient, and MS method.

### Culturing of mESCs and differentiation

mESCs (SCR012, strain 129/S6/SvEv, MILLIPORE, Billerica, Massachusetts, USA) were maintained on mitomycin C-treated MEF feeders in Dulbecco’s modified Eagle’s medium supplemented with 16 % ESC-tested fetal bovine serum (Hyclone, Logan, Utah, USA), 0.15 mM α-monothioglycerol, 2 mM l-glutamine, 0.1 mM nonessential amino acid, 10 mM HEPES (Sigma-Aldrich, St. Louis, Missouri, USA), 1000 U/ml recombinant leukemia inhibitory factor (LIF, Chemicon, Temecula, California, USA), and 100 U/ml penicillin/streptomycin.

For in-vitro differentiation, 2 × 10^6^ feeder-depleted ESCs were aggregated in 10 cm ultra-low attachment plates (Corning, New York, USA) in embryonic stem differentiation medium without LIF. Embryoid bodies were collected at day 0 (D0), day 2 (D2), and day 4 (D4).

### Quantitative real-time PCR

RNA was isolated from 1 × 10^6^ ESCs or 200 embryonic bodies using the RNeasy Plus kit (QIAGEN, Duesseldorf, Germany). First-strand cDNA synthesis was made with the SuperScript III First-Strand Synthesis SuperMix (Invitrogen, Carlsbad, California, USA). The relative expression of each gene was determined by normalizing to Gapdh mRNA level. Quantitative RT-PCR was performed with SYBR Green Master Mix (Bio-Rad, Hercules, California, USA) on an Eppendorf Mastercycler ep realplex2 s (Eppendorf, Hamburg, Germany). Primers for tested genes are from [17, 18].

### LC-MS/MS for tryptic histone peptides from mESCs

Intact histones were extracted from mESCs using a conventional acid extraction method [19]. A total of 100 μg freeze-dried histones were digested with trypsin (V511C; Promega) at a substrate/enzyme ratio of 200:1 for 40 minutes at 37 °C. Tryptic peptides were analyzed using a Shimadzu HPLC system (Shimadzu, Kyoto, Japan) interfaced to an LTQ-Orbitrap XL instrument run in positive ion mode. The LC was operated in a 25 cm analytical column (1 mm inner diameter, ZORBAX 300SB-C18, 5 μm, 300 Å; Agilent, Palo Alto, California, USA) with a 1.7 cm precolumn (1 mm inner diameter, ZORBAX Stablebond, C18, 5 μm, 300 Å; Agilent). Here, Solvent A was 5 % acetonitrile (Fisher Scientific) with 0.05 % trifluoroacetic acid (TFA) in ddH_2_O, and solvent B was 90 % acetonitrile with 0.05 % TFA. A total of 20 μg tryptic sample was injected into solvent A at a flow rate of 50 μl/minute. The gradient was 5–45 % solvent B from 5 to 45 minutes. The mass spectrometer was operated in the data-dependent mode to automatically switch between MS and MS/MS acquisition. The full-scan MS spectra were acquired in Orbitrap with a resolution of 60,000 FWHM. The full MS mass range was 250–2000, AGC target 1,000,000, and maximum injection time 500 milliseconds. The five most intensive ions with a parent 3+ charge precursor of K27-R40me5 (501.9755) were isolated for fragmentation in the LTQ using CID (normalized collision energy 35 %). Singly charged precursor ions were excluded, and the dynamic exclusion time was not enabled.

### Western blotting

To examine the global level of H3K27me3 in ES cells at D0, D2, and D4, 1 μg extracted bulk histones from these cells were resolved by 15 % SDS-PAGE respectively. H3K27me3 specific antibody (07–449; Upstate Biotechnology, Lake Placid, New York, USA) was first used, and then striped for H3 antibody (ab1791; Abcam, Cambridge, UK) detection as the loading control.

### Quantification by SIMC

Database searching for MS/MS spectra was performed against histone sequences in the UniProt database (20 January 2009) with 29 entries using SEQUEST within Bioworks 3.3.1 SP1 software (ThermoFisher Scientific) [20]. Five missing cleavages were allowed and mass tolerance of 20 ppm was set for precursor ions. Variable modifications included protein N-terminal acetylation, (mono, di, and tri)methylation and acetylation of lysine, (mono and di)methylation of arginine, phosphorylation of serine and threonine, and oxidation of methionine. Initial acceptance criteria for the match required a mass error of precursor less than 0.01 Da, and the search results from peptides of interest were then manually verified using Bioworks Browser software.

The quantification method for SIMC is the following: extract the matched MS/MS spectra from the raw data using Xcalibur 2.0.7 software (ThermoFisher Scientific); calculate the theoretical *m/z* values of a series of specific y ions for individual isobarically modified peptide according to the monoisotopic mass from MS-Product (UCSF, San Francisco, California, USA); search for matched y ions from the MS/MS spectral data according to the theoretical values with a mass tolerance of 10 ppm for high-resolution Orbitrap data or 0.4 Da for LTQ data, and select one with the smallest mass error for a specific y ion (there is no signal-to-noise threshold for the selection of ions); sum up the intensity of all the matched y^2+^ ions specific for an isoform, which represents the quantity of the isoform; and calculate the percentage of each isoform based on the quantity of every isoform. The fragments with the minimal mass errors were selected and their information was listed in a table including matched type of isobaric modification, MS/MS scan number, corresponding retention time, intensity, *m/z* value, and mass error.

For quantification of H3K27-R40me5 and H3D123-R128 peptides, conventional SIC was used. The SIC values of modified peptides were obtained by summing up individual SIC values from all charge states: 1–4+ for H3K27-R40me5 peptides, and 1–2+ for H3D123-R128 peptides with a mass tolerance of 10 ppm for Orbitrap data. By multiplying the total amount of the H3K27-R40me5 and the constituent percentage of each isoform determined by SIMC, we quantified the abundance of K27me2-K36me3 and K27me3-K36me2 isoforms. The abundances of K27me2-K36me3 and K27me3-K36me2 isoforms were normalized to the SIC values of H3D123-R128 peptides to acquire their relative abundance. All experiments in this study were repeated three times and the deviation number was the standard deviation (SD). Data are expressed as mean ± SD. Comparisons involving three mESC groups were performed with one-way analysis of variance.

## Results and discussion

### Comigration of H3K27me3-K36me2 and H3K27me2-K36me3-containing K27-R40 peptides in RP-HPLC

**Fig. 1:**
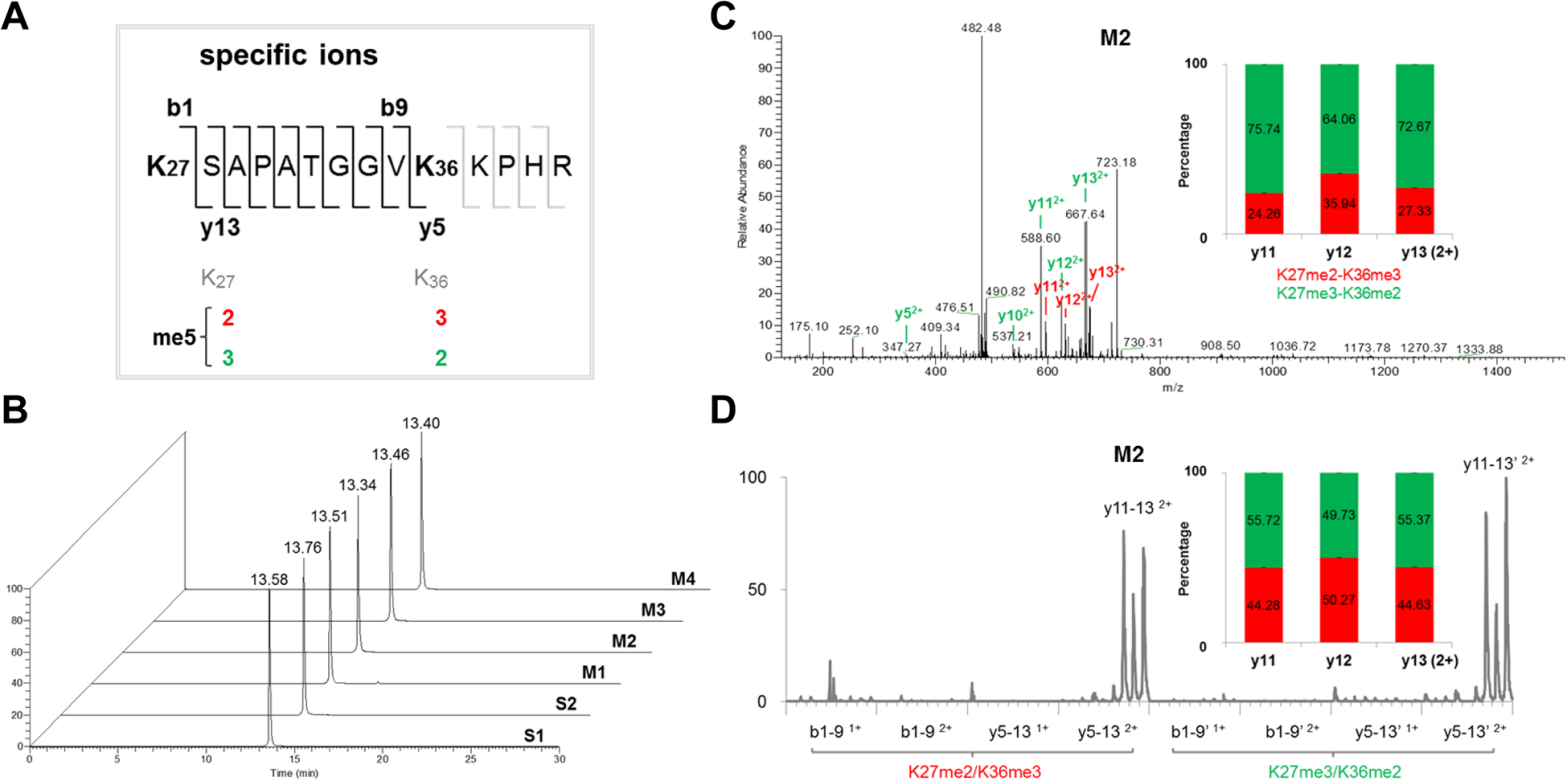
Characteristics of H3K27me3-K36me2 and H3K27me2-K36me3-containing peptides. **a** Schematic representation of isobaric pentamethylation of histone H3K27-R40 peptides and their specific ions. **b** Comigration of H3K27me3-K36me2 and H3K27me2-K36me3-containing peptides on RP-HPLC. Two synthetic isobarically modified peptides of H3K27-R40, S1 (K27me2-K36me3) and S2 (K27me3-K36me2), and their mixtures, M1 (3:1), M2 (1:1), M3 (1:3), and M4 (1:6), were subjected to LC-MS analysis. **c** Strongest tandem mass spectrum of the M2 sample. Two types of specific y^2+^ ions generated by each isoform were marked with different colors (K27me2-K36me3, *red*; K27me3-K36me2, *green*). Quantification was based on the intensity of two types of y11–y13^2+^ ions in the single spectrum. **d** The intensity of two types of b^1+^, b^2+^, y^1+^ and y^2+^ specific ions in a series of tandem mass spectra of M2 was displayed as a chromatographic profile. Quantification was based on the peak area of two types of y11–y13^2+^ ions

RP-HPLC was applied to separate H3K27me3-K36me2 and H3K27me2-K36me3-containing peptides since they are isobarically modified peptides with the same molecular weight. Two peptides of H3K27-R40me5, S1 (K27me2-K36me3) and S2 (K27me3-K36me2) (Fig. 1a), mimicry of the enzymatic products of histone H3, were synthesized and tested by RP-HPLC. When each peptide was examined by LC-MS/MS, the retention time of S1 was around 13.58 minutes and of S2 was around 13.76 minutes (Fig. 1b), indicating that both peptides had similar chromatographic behavior. To examine whether two peptides coelute in mixtures, four different ratios of two peptides (M1–M4) were mixed and examined under the same experimental conditions. Despite the four weight ratios of the peptides in the mixture, only one single peak with a similar retention time to the pure forms was observed at around 13.34–13.52 minutes (Fig. 1b). These results indicated that H3K27me2-K36me3 and H3K27me3-K36me2-containing peptides were coeluted in RP-HPLC, and could not be separated and quantified for the same molecular weight at the first MS level, which was consistent with published data [12, 21, 22].

### Examination of specific ions for H3K27me3-K36me2 and H3K27me2-K36me3-containing peptides

At the MS level, H3K27me3-K36me2 and H3K27me2-K36me3-containing peptides produced the same 1–4+ precursor ions, namely no different intensity distribution between individual and mixed peptides (predominantly 3+ and 4+ charged precursors). Although inseparable by RP-HPLC and indistinguishable at the MS level, H3K27me3-K36me2 and H3K27me2-K36me3-containing peptides are able to produce fragment ions with different masses by MS/MS (Additional file 1). As indicated in Fig. 1a, y5–13 and b1–9 ions generated by H3K27me3-K36me2 and H3K27me2-K36me3-containing peptides will have different masses and therefore are specific ions for each isoform. For example, y13 ions with two positive charges (y13^2+^) generated by H3K27me3-K36me2 and H3K27me2-K36me3-containing peptides have masses of 667.3886 and 674.3964, respectively. This difference indicates that we can target the same precursor to generate the MS/MS spectra, and then may employ the different specific ions to determine the constituent percentage of the two isoforms. Theoretically, the 3+ precursor should give rise to a series of fragment ions with 1+ and 2+ charges, while the 4+ precursor can form 1–3+ charged fragment ions. In the CID fragmentation of bottom-up MS approach, y and b fragment ions with 1+ and 2+ charges are commonly accepted for the assignment of peptide sequence [20, 23, 24], so we target the 3+ precursor of the pentamethylated K27-R40 peptides for subsequent fragmentation and specific ion analysis.

To test whether we can detect these specific ions, we performed a LC-MS/MS experiment using the two synthetic peptides, and targeted the 3+ charged precursor of the pentamethylated H3K27-R40 peptides for subsequent fragmentation. Overall, whatever the composition of single or mixed peptide samples, a series of y ions with two positive charges (y ^2+^) were predominantly detected from the 3+ charged precursor, and other fragment ions such as y^1+^, b^1+^, and b^2+^ were not (Fig. 1c, d; and Additional file 1). Among these, y11–13^2+^ ions were more intense for the appearance of the proline residue [25, 26]. These results indicate that the H3K27me3-K36me2 and H3K27me2-K36me3-containing peptides are coeluted and cofragmented in the LC-MS/MS determination.

### SIMC quantification of H3K27me3-K36me2 and H3K27me2-K36me3-containing peptides by MS/MS

To examine how we can use these specific ions to know the constituent percentage of H3K27me3-K36me2 and H3K27me2-K36me3-containing peptides, we performed LC-MS/MS of a mixture containing an equal amount of the two peptides (M2). Firstly, we calculated the intensity ratios of individual specific y ion pairs in one single fragmentation scan. However, even in the scan acquired when the precursor was strongest, only two types of y8^2+^ and y10–13^2+^ were detected simultaneously. As shown in Fig. 1c, *m/z* values of 588.60, 624.08, and 667.64 corresponded to y11–13^2+^ ions for H3K27me3-K36me2-containing peptide, and *m/z* values of 595.48, 631.03, and 674.43 corresponded to y11–13^2+^ ions for H3K27me2-K36me3-containing peptide, respectively. From the intensity of y11–13^2+^ ion pairs, the constituent ratios of H3K27me3-K36me2 and H3K27me2-K36me3-containing peptides were calculated to be 75.74:24.26 for y11^2+^, 64.06:35.94 for y12^2+^, and 72.67:27.33 for y13^2+^, respectively. These data were not consistent with the original compositions (Fig. 1c). In addition, the intensity ratios of all three y ion pairs varied between different scans (data not shown). The variation of the intensity of y11–13^2+^ ions indicates unstable fragmentation efficiency among individual scans, which makes quantification using one ion pair in one single scan infeasible.

To examine whether the cumulative intensity of specific ions from multiple scans can be used to determine the relative abundance of each peptide, we generated the intensity curves of y11–13^2+^ ions from multiple scans along the elution time, as well as all the other 1+ and 2+ charged specific y and b ions (Fig. 1d). Consistent with the results in the individual scan, not all specific ions were detectable even in multiple MS/MS scans, and y11–13^2+^ ions were present predominantly as revealed by the intensity curves (Fig. 1d). We designated the peak area of the intensity curve as the cumulative intensity of each ion and calculated the ratio of each ion pair. The peak area ratio of each ion pair still varied significantly (Additional file 2). As displayed in Fig. 1d, the ratio of y11^2+^ ions specific for H3K27me3-K36me2 and H3K27me2-K36me3-containing peptides was 55.72:44.28, whereas the ratio was 49.73:50.27 for y12^2+^ and 55.37:44.63 for y13^2+^, respectively. These results indicated that the quantification was also not reliable using the ratio of the cumulative intensity of one ion pair from multiple scans; even using the most intense y11–13^2+^ ion pairs, there were still small variations for the quantification.

**Fig. 2:**
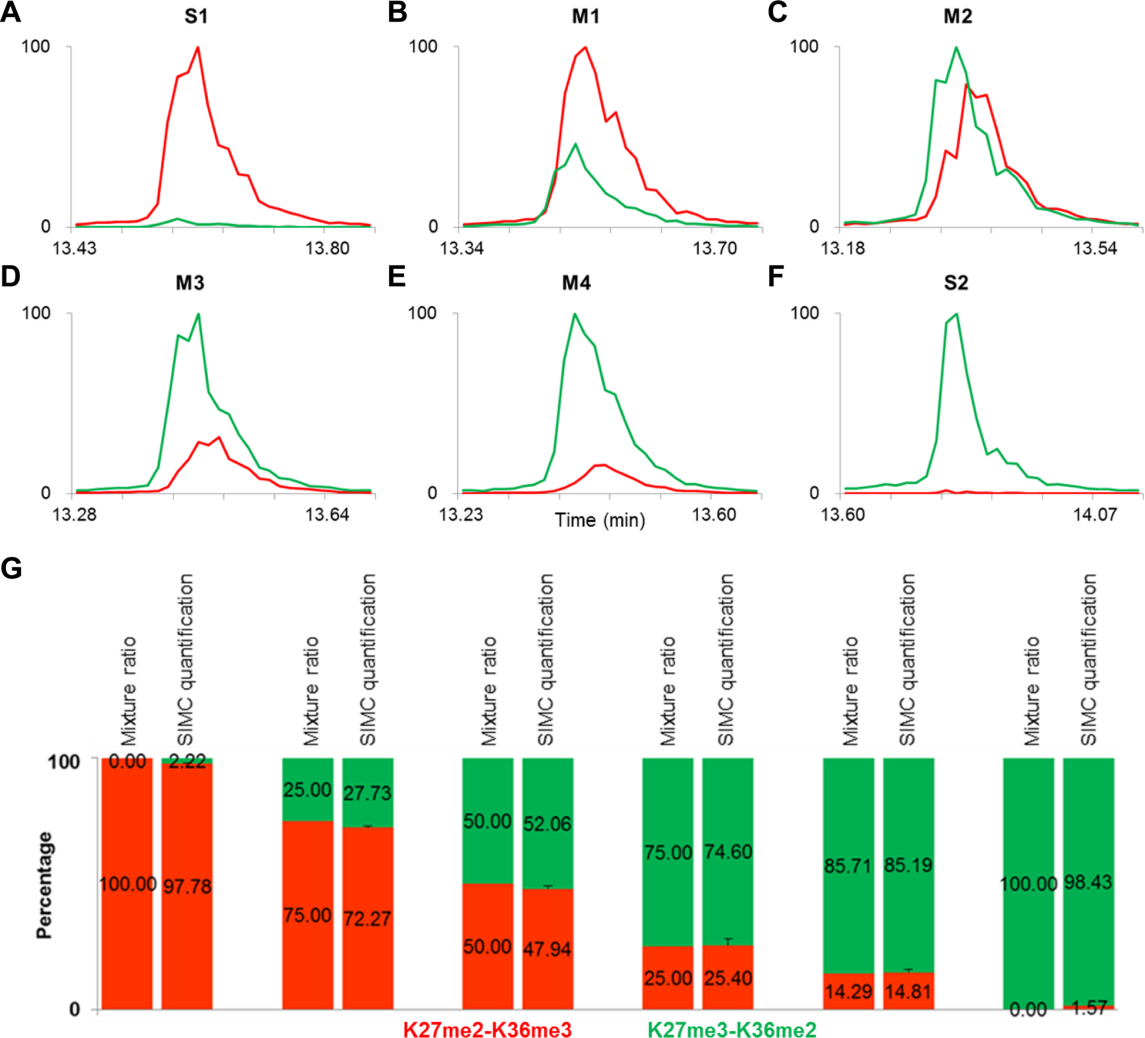
SIMC quantification of H3K27me3-K36me2 and H3K27me2-K36me3-containing peptides by MS/MS. S1 (K27me2-K36me3) and S2 (K27me3-K36me2) of H3K27-R40me5, and their mixtures M1–M4, were subjected to LC-MS/MS analysis. **a–f** The intensity of two types of y5–13^2+^ in multiple MS/MS spectra of all samples was summed up and displayed as a SIMC profile. **g** SIMC quantification of K27me3-K36me2 (*green*) and K27me2-K36me3 (*red*) in H3K27-R40me5. The MS experiment was repeated three times and the error bars represent the SD. *SIMC* specific ions of isobaric modification chromatogram

To avoid the bias of ion pair selection, we test whether the sum-up of the cumulative intensities of a series of specific ions can represent the total amount of each peptide more accurately. After calculating the ratios of b^1+^, b^2+^, y^1+^, and y^2+^ specific ions, respectively, we found that only the ratio from y^2+^ ions almost matched the real peptide composition in M2. The mean percentage calculated from y^2+^ specific ions (y5–13^2+^) was 52.06:47.94 (Fig. 2g). Moreover, the sum-up peaks of two types of y5–13^2+^ ions from multiple scans could reflect the amount of H3K27me3-K36me2 and H3K27me2-K36me3-containing peptides in M2, and also matched the profile of their precursors along the elution time (Fig. 2c).

To confirm this conclusion, H3K27me3-K36me2 and H3K27me2-K36me3-containing peptides were mixed at different ratios of 1:0, 6:1, 3:1, 1:3, and 0:1, and subjected to LC-MS/MS. The calculated percentages from the sum-up peaks of two types of y5–13^2+^ ions from multiple scans were 98.43:1.57, 85.19:14.81, 74.60:25.40, 27.73:72.27, and 2.22:97.78, corresponding to the theoretical values of 100:0, 85.71:14.29, 75:25, 25:75, and 0:100, respectively, with a variance of less than 2.71 % (Fig. 2g). These results indicated that the constituent percentage of H3K27me3-K36me2 and H3K27me2-K36me3-containing peptides could be represented by the sum-up intensity of y5–13^2+^ specific ions from multiple scans. Because the quantitative peaks depicted the intensity changes of specific ions with the chromatographic elution of their common precursors, this method was named SIMC.

To ensure that specific ions used for quantification were accurately assigned, high mass accuracy MS/MS spectra on Orbitrap were conducted under the same experimental conditions. The calculated percentages were 99.84:0.16, 89.01:10.99, 80.32:19.68, 57.41:42.59, 31.61:68.39, and 0.97:99.03, corresponding to the theoretical values of 100:0, 85.71:14.29, 75:25, 50:50, 25:75, and 0:100, respectively, with the variance of less than 0.91 % (Figure S3G in Additional file 3). As displayed in Additional files 3 and 4, the cumulative peaks were more regular and the quantitative percentage from Orbitrap data could also reflect a change in the proportion of H3K27me3-K36me2 and H3K27me2-K36me3-containing peptides compared with those from LTQ data (Figs. 1d and 2). However, the values from LTQ were much closer to the loaded ratio (Figure 2g; and Figure S3G in Additional file 3). Although Orbitrap can provide a high-resolution spectrum, the method is insensitive and the data acquisition is relatively slower, and therefore fewer MS/MS spectra and less specific ions make the SIMC results from Orbitrap a little further from the loaded ratio (Figure S1B in Additional file 1, Figure S2B in Additional file 2, and Additional files 3 and 4). This observation indicated that the high-accuracy Orbitrap at the first MS level could minimize the impurity interference, making the relatively low-accuracy MS/MS spectra by LTQ sufficient for accurate quantification. Also, this result implies that the SIMC approach can have a wide potential application. Considering that LTQ is commonly used for MS/MS spectra for higher sensitivity and scan speed than Orbitrap, we applied Orbitrap-LTQ MS/MS in this study, with Orbitrap at the first MS level for the accuracy of the precursor and LTQ at the MS/MS level for more information of the fragment ions. To further assess the accuracy of the SIMC method, we performed the crucial validation experiment in a complex biological background. The M2 sample was mixed with the tryptic *Escherichia coli* proteins, which was known without any histones, and then analyzed by LC-MS/MS. Finally, the calculated SIMC percentage was 55.20:44.80, closer to the loaded ratio of M2, with a variance of 1.28 %. This result further validated the robustness of the SIMC method. Since histones are highly conserved proteins, the target K27-R40 peptides of H3.1 and H3.2 all have the same sequence either in yeast or mammalian cells. Undoubtedly, the pre-existing histone can affect the result for the same H3 sequence and modification, and thus it is necessary to avoid the contamination of any yeast or other mammalian cells.

### Effects of tryptic digestion on the quantification of H3K27me3-K36me2 and H3K27me2-K36me3-containing peptides

Quantification of the H3K27me3-K36me2 and H3K27me2-K36me3 modifications requires the generation of a H3 peptide containing both K27 and K36 residues. Trypsin and Arg-C are the most frequently used proteases in the bottom-up MS method to produce H3K27-R40 peptides containing the target modifications. Arg-C specifically cleaves at the C-terminus of arginine residues and its digestion of H3 can generate the K27-R40 peptide that contains K27 and K36 residues [15]. Trypsin, however, can cleave both arginine and lysine residues except with an immediate adjacent proline (P) on the carboxyl side of the two residues. For the methylated residues, trypsin’s digestion efficiency decreased with the increase of methyl groups. Generally, trypsin can cut monomethylated lysine, but not dimethylated or trimethylated lysine [23, 27]. Hence, tryptic digestion of histone H3 will also generate K27-R40 peptides. In addition, compared with the low efficiency of Arg-C, trypsin can quite effectively digest hypomethylated K27-R40 peptides into smaller ones, such as K27-K36 and S28-R40. As a result, trypsin digestion can enrich the H3K27-R40 peptides with a high degree of methylation, including pentamethylation. Considering the small amount of pentamethylation at H3K27 and H3K36 in the mammalian cells examined, and the priority of more abundant peptides for MS/MS detection, we expected to improve the relative abundance of the pentamethylated K27-R40 peptides by selection of trypsin instead of Arg-C, so as to acquire as many MS/MS spectra as possible for SIMC quantification.

**Fig. 3:**
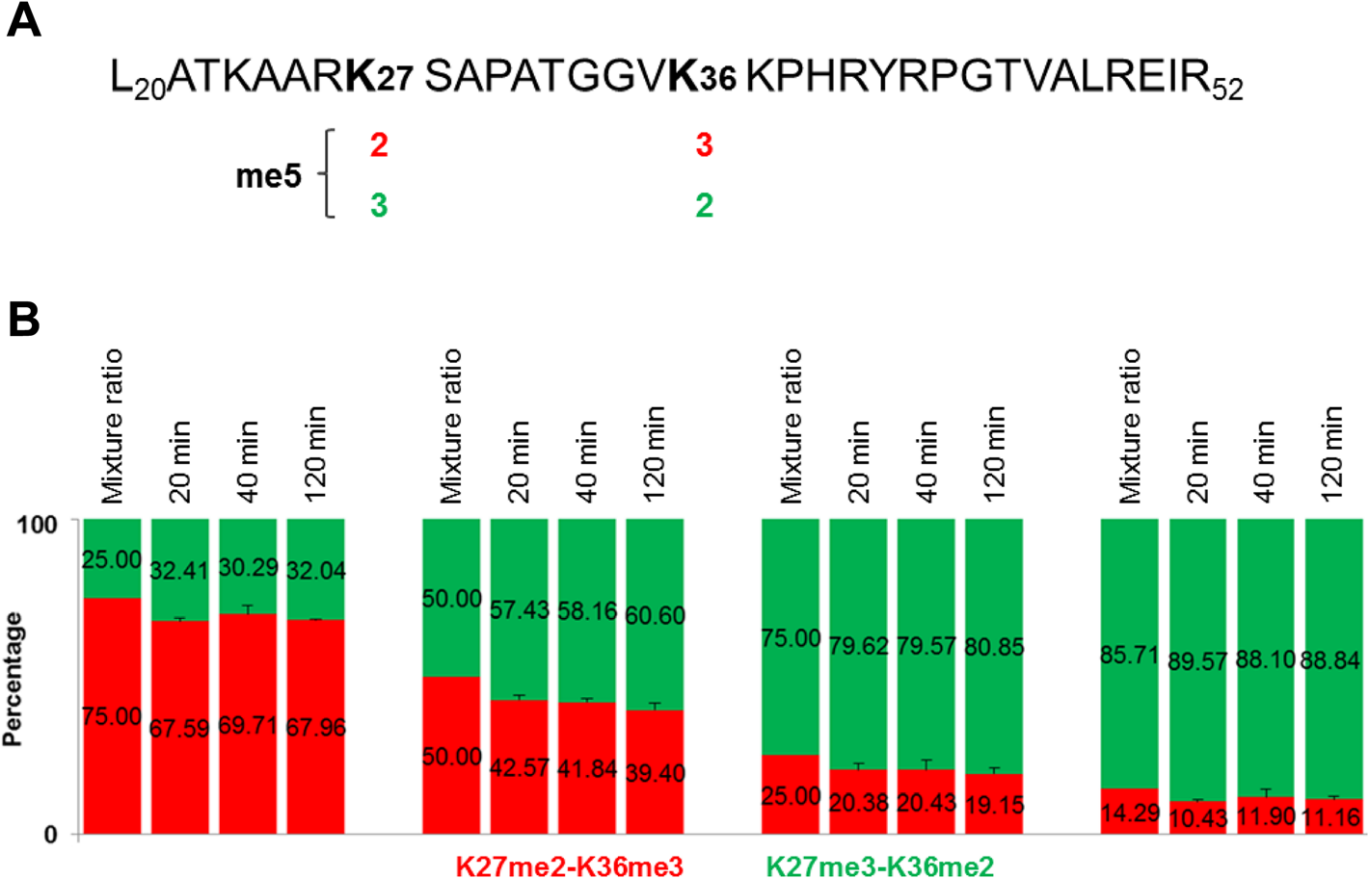
SIMC quantification of tryptic K27me2-K36me3 and K27me3-K36me2-containing H3L20-R52me5 peptides. **a** Schematic representation of the isobaric H3L20-R52me5 peptides. **b** SIMC quantification of K27me3-K36me2 (*green*) and K27me2-K36me3 (*red*) in H3L20-R52me5. L1 (K27me2-K36me3) and L2 (K27me3-K36me2) of H3L20-R52me5 with different ratios (3:1, 1:1, 1:3, and 1:6) were digested by trypsin for 20, 40, and 120 minutes, respectively, and were analyzed by LC-MS/MS. The MS experiment was repeated three times and error bars represent the SD

To determine whether trypsin digestion may affect the quantification of H3K27me3-K36me2 and H3K27me2-K36me3-containing peptides, we synthesized two longer peptides of H3L20-R52me5, L2 (K27me3-K36me2) and L1 (K27me2-K36me3), mimicry of the intact histone H3 carrying the two combinatorial modifications (Fig. 3a). We then mixed the two peptides at different ratios of 6:1, 3:1, 1:1, and 1:3, and performed trypsin digestion. The digestion time is decided by enzyme efficiency and target peptides. It is known that trypsin digestion is quite efficient for histone proteins which are rich in lysine and arginine residues [28]. Therefore, trypsin partial digestion has been frequently used for the characterization of histone modification [22, 29, 30]. Since the methyl groups of lysine can partially hinder the trypsin digestion [28], and to reduce the possible cleavage for dimethylated or trimethylated lysine as much as possible, we performed trypsin in-solution digestions for a short time of 20, 40, and 120 minutes, respectively.

The digested peptide mixtures were first examined using matrix-assisted laser desorption ionization (MALDI) to evaluate the digestion efficiency. Although there existed little insufficient digestion by MALDI in all digested samples, the content of underdigested products or peptides longer than H3K27-R40me5 was too low to be detected by LC-MS/MS. This observation suggests that trypsin has considerably high efficiency to produce the target pentamethylated H3K27-R40 peptides. We then detected these digested samples by LC-MS/MS, and quantified them by SIMC for the constituent percentage of the isobarically pentamethylated H3K27-R40 peptides. The calculated percentages were similar among different digestion times (20, 40, and 120 minutes, respectively), with average values of 88.84:11.16, 80.01:19.99, 58.73:41.27, and 31.58:68.42, respectively, and were able to reflect the change in the mixing proportion of two long peptides (Fig. 3b; and Additional file 5). Although there was little overall synchronous decline to the H3K27me2-K36me3 isoform in all mixtures, to the extent of about 5 %, this could be explained by the original concentration difference of the two long peptides or the weak preference for H3K27me2-K36me3 by trypsin digestion (Fig. 3b; Additional file 5). These results indicate that trypsin digestion does not affect the SIMC quantification of H3K27me3-K36me2 and H3K27me2-K36me3-containing peptides.

Among the investigated digestion times, the SIMC values for 40 minutes were a little closer to the theoretical mixture ratio of two H3K27me3-K36me2 and H3K27me2-K36me3-containing H3L20-R52 peptides (Fig. 3b). Therefore, we chose this condition for trypsin digestion of intact histones.

### Quantitative analysis of H3K27me3-K36me2 and H3K27me2-K36me3 in mESCs and differentiated cells

The important regulatory interplay between H3K27me3 and H3K36me3 in the embryonic development suggests a possible role for other higher methylation states of these two sites. To determine whether cis-existing H3K27me3-K36me2 and H3K27me2-K36me3 play a role in the process, we applied SIMC at MS/MS level combined with SIC at MS level to quantify the two modifications in mESCs and differentiated cells. Histones from mESCs (D0) and those induced to differentiate for 2 days (D2) and 4 days (D4) (Additional file 6) were digested by trypsin and analyzed by LC-MS/MS.

Through SIC of the precursors at MS level, we calculated the percentages of H3K27-R40me0–6 (Additional file 7). As expected, the hypomethylated H3K27-R40me0–3 peptides were decreased, while the pentamethylated K27-R40 peptides were enriched to 10.77–17.77 % by trypsin (1:200, 40 minutes) digestion compared with ~5 % by Arg-C (Additional file 7) [15, 31]. Consistent with the published data [15, 16], H3K27-R40me5 peptide was more abundant than H3K27me3-K36me3 in ESCs. Worthy of note is that no peptides containing K27 and K36 longer than H3K27-R40 were detected, while only acetylation coexisting with methylation was observed and present with one acetyl group on K27 and zero to three methyl groups on K36 on the H3K27-R40 peptide. The short H3K27-K36 peptides in the trypsin-digested histone samples were present with zero to three methyl groups on K27 and no methyl group on K36, indicating that trypsin digestion was able to enrich the target pentamethylated H3K27-R40 peptides for MS quantification.

**Fig. 4:**
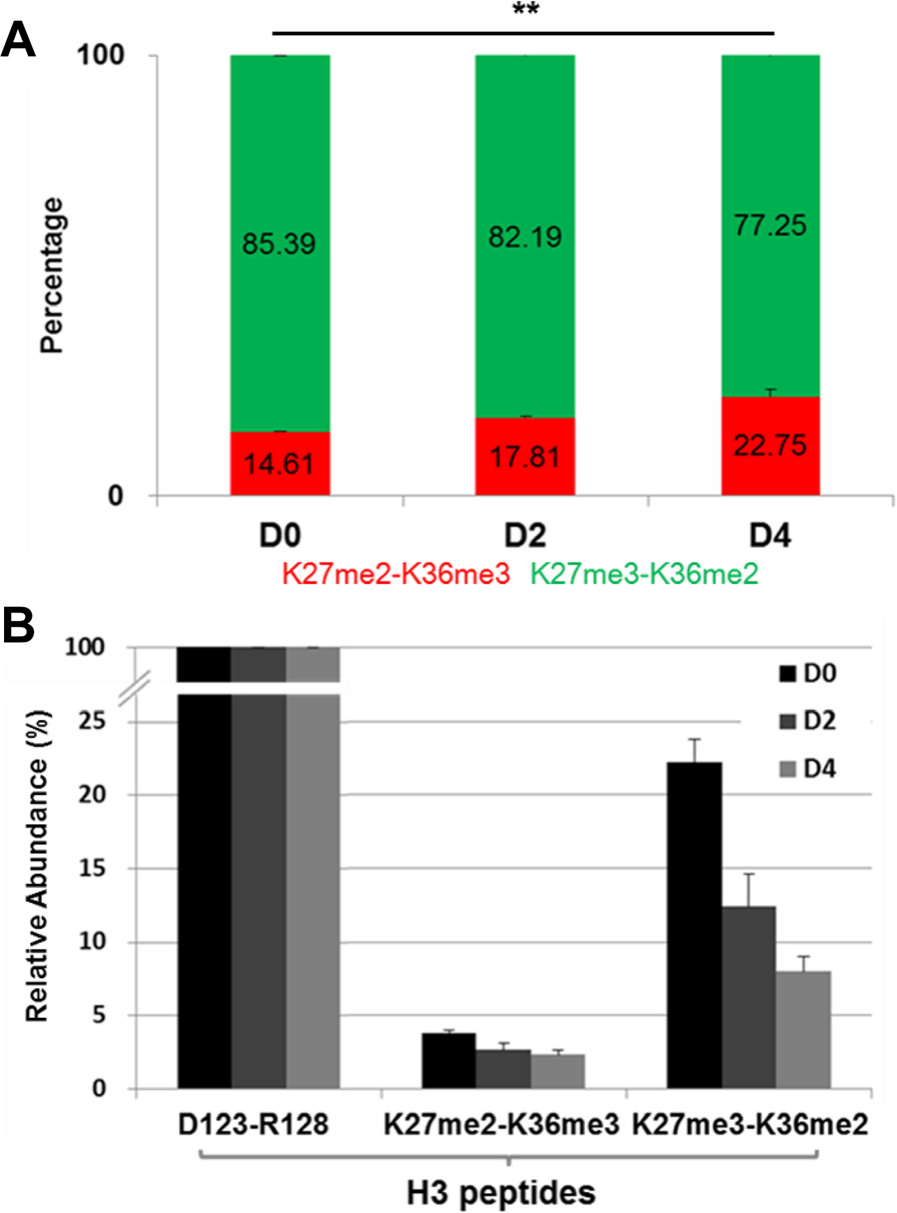
Quantification of isobaric H3K27-R40me5 in mESCs and differentiated cells. **a** SIMC quantification of K27me3-K36me2 (*green*) and K27me2-K36me3 (*red*) isoforms of H3K27-R40me5 in histones from mESCs (D0) and at differentiation day 2 (D2) and day 4 (D4) induced by LIF withdrawal. **b** Relative abundance of each isoform of H3K27-R40me5 in mESCs and differentiated cells normalized to the H3D123-R128 peptide. The levels of H3K27-R40me5 and H3D123-R128 peptides were determined by SIC, and the amount of K27me2-K36me3 and K27me3-K36me2 isoforms was calculated by multiplying the total level of the H3K27-R40me5 by SIC and the constituent percentage of each isoform by SIMC. The MS experiment was repeated three times and the error bars represent the SD. ** *P* <0.01

Using the SIMC approach, the constituent percentages of H3K27me3-K36me2 and H3K27me2-K36me3 in mESCs and differentiated cells were calculated. In mESCs, H3K27me3-K36me2 was the dominant form, comprising 85.39 % of the pentamethylated H3K27-R40 population. Upon ESC differentiation, the percentage of this modification decreased to 82.19 % at D2 and to 77.25 % at D4, respectively, with a variance of less than 1.48 % (*P* = 0.000512, Fig. 4a; and Additional file 8).

To determine whether the decrease in percentage of H3K27me3-K36me2 modification was caused by a decrease of its own level or by an increase of H3K27me2-K36me3 level, and also to avoid the influence of tryptic digestion for the hypomethylated K27-R40 peptides into smaller ones, the abundance of two isoforms was normalized to a H3D123-R128 peptide (a constitutive and modification-free tryptic peptide of H3). The results show that H3K27me3-K36me2 was the dominant pentamethylated form at H3K27 and H3K36 residues in mESCs, and the relative abundance of H3K27me3-K36me2 still significantly decreased upon mESC differentiation, while the level of H3K27me2-K36me3 almost remained unchanged (Fig. 4b). In addition, the decrease of H3K27me3-K36me2 isoform was co-occurred with a global downregulation of K27me3 level observed by western blot (Additional file 9) [32, 33]. These results indicate that the cis-existence of H3K27me3-K36me2 modification is the dominant pentamethylated form at H3K27 and H3K36 residues in mESCs and is downregulated upon differentiation, suggesting that it may assume the main regulatory function in ESC differentiation.

The higher methylation states at H3K27 and H3K36 residues play a crucial role in the embryonic development [10, 11]. Since the residues of H3K27 and H3K36 are close at the short N-termini of histone H3, they will more probably act as a combinatorial methylation pattern. The small but significant amount of histone pentamethylation at H3K27 and H3K36 detected by MS indicates that the cis-existing H3K27me3-K36me2 and/or H3K27me2-K36me3 may make a regulatory function in ESC differentiation [11, 12]. However, it has been found that the two modifications rarely colocalized at the same genomic locus, consistent with their opposing functions [8, 9]. The discordant results relate to the technology: the antibody-dependent technologies, such as genome-wide ChIP-Seq and western blot, are good for one modified state of one single residue, but cannot reveal the combinatorial modifications of the same histone molecule; while high-resolution MS is able to provide information about the combinatorial modifications, and to usher a new direction for deciphering the “histone code” [34–36]. For the same sequence and the same number of modified groups but different sites, the H3K27me3-K36me2 and H3K27me2-K36me3-containing peptides are isobarically modified peptides, which remain a big challenge for MS detection.

To determine the colocalization of two opposing methylations at H3K27 and K36 residues, we fully used the characteristics of comigration in RP-HPLC, codetection as the same precursor, and cofragmentation for two types of specific ions, and established a simple, label-free, bottom-up MS method—called SIMC—for the systematic quantification of H3K27me2-K36me3 or H3K27me3-K36me2 isoforms. As a result, the intensity variation of specific ions among individual fragmentations could be minimized by the LC stability. The selection bias of specific ions could be avoided by a series of specific ions, and thus the accurate constituent percentage of each isobaric form could be achieved by the sum-up of the cumulative intensities of a series of specific ions along the multiple consecutive scans. Because the SIMC quantification is based on a series of specific ions for each isobaric modification from multiple MS/MS spectra, the amount of precursors is the premise for the acquisition of subsequent MS/MS spectra. By choice of trypsin digestion, we decreased the H3K27-R40 peptides with relatively lower methylation degree and enriched our target, the small amount of pentamethylation at H3K27 and H3K36 in ESCs, which ensured the acquisition of enough MS/MS spectra for SIMC quantification. Generally, it is necessary to acquire enough spectra to depict the outline of the LC peak, so as to guarantee the chromatogram for the selected ion. In this study, all SIC levels were calculated from 22 MS spectra, and all SIMC values were calculated from more than six MS/MS spectra along the LC profile of the precursor, so that the quantification was stable and reproducible.

The SIMC is a robust method of quantification. To establish and test the method, we synthesized two peptides of H3K27-R40me5, S2 (K27me3-K36me2) and S1 (K27me2-K36me3), and designed six mixing ratios of the two isoforms (1:0, 6:1, 3:1, 1:1, 1:3, and 0:1), with a dynamic range from 0 to 100 %. The quantitative values by SIMC were more than 97.8 % for the pure peptide samples, and also were able to accurately quantify the proportion of two peptides in all mixtures, with a variance of less than 2.71 % (Fig. 2). In this study, although the relatively small mass tolerance was set for matched ions (0.4 Da for LTQ data), some interference ions with low intensity appeared which lowered the calculated ratios for pure synthetic peptides (<100 %). To determine whether trypsin digestion will affect the SIMC quantification of H3K27me3-K36me2 and H3K27me2-K36me3-containing forms, two long peptides of H3L20-R52me5, L1 (K27me2-K36me3) and L2 (K27me3-K36me2), were synthesized and mixed as the short ones (H3K27-R40me5). After trypsin digestion for 20, 40, and 120 minutes, the SIMC values were still able to reflect the change in the mixing proportion of two long peptides, with a variance of less than 3.20 % (Fig. 3; and Additional file 5). Although the total levels of H3K27-R40me5 from SIC were only 10.77–17.77 %, the constituent percentages of H3K27me3-K36me2 and H3K27me2-K36me3 were calculated to be 77.25–85.39:14.61–17.81, with a variance of less than 1.48 % (Fig. 4a; and Additional files 7 and 8).

The sensitivity limit of SIMC was further studied by diluted M2 samples (Additional file 8). In a 500-fold dilution of M2 (i.e., 0.15 ng total synthetic peptides loaded), the SIMC percentage was observed to be 53.30:46.70, with a variance of 1.26 %, indicating that the sensitivity limit of the SIMC method was 50 pg/μl (100 pmol) for H3K27-R40me5 peptides in our Orbitrap-LTQ instrument (Additional file 10). Worthy of mention is that the SIMC approach is based on the tandem spectra, and therefore the sensitivity of the method mainly depends on the precursor concentration at the MS level. Obviously, the SIMC approach is accurate, sensitive, and stable for systematic quantification of the isobaric H3K27me2-K36me3 and H3K27me3-K36me2 modifications.

Notably, the peak area ratio of each ion pair for H3K27me2-K36me3 and H3K27me3-K36me2 isoforms varied significantly (Additional file 2). Using a series of main specific ions (y5–13^2+^ ions), we could eliminate the ion selection variation as much as possible and achieve accurate and stable quantification (Figs. 2 and 3). Therefore, we can infer that only three discriminative b and/or y fragment ions for each species were used: b1^1+^, y11^2+^, and y13^2+^ for H3K27me2-K36me3; and b8^1+^, b9^1+^, and y11^2+^ for H3K27me3-K36me2, the quantification may be unreliable for the selection of different specific ions [37]. For the same reason, if the isobaric modifications are distributed at adjacent residues, the quantification may be influenced by the limitation of a specific ion number.

Considering the principle of the method, it is perceivable that the SIMC can be used to examine the other isobaric modifications, such as acetylation, and to dissect the coregulation of more combinatorial modifications, which will contribute greatly to interpretation of the “histone code”.

## Conclusions

By establishing a label-free, bottom-up mass spectrum method, SIMC, we not only revealed the cis-existence of H3K27me3-K36me2 in mESCs, but also suggested that this combinatorial histone modification may assume a specific regulatory function during differentiation.

## Additional files

Additional file 1: Figure S1. Showing the strongest tandem mass spectra of S1 (H3K27me2-K36me3), S2 (H3K27me3-K36me2), and M2 (containing an equal amount of S1 and S2 peptides) by LTQ **A** and by Orbitrap **B**. (DOCX 123 kb) Additional file 2: Figure S2. Showing quantification of H3K27me3-K36me2 and H3K27me2-K36me3-containing peptides in M2 based on the peak area of individual specific ion by LTQ **A** and by Orbitrap **B**. All 1+ and 2+ charged b1–9 and y5–13 specific ions were examined in multiple tandem mass spectrum of M2, a mixture containing an equal amount of the two peptides of H3K27-R40me5, S1 (K27me2-K36me3) and S2 (K27me3-K36me2). The peak area of the intensity curve in Fig. 1d and Additional file 4 was designated as the cumulative intensity of each ion to calculate the ratio of each ion pair. (DOCX 204 kb) Additional file 3: Figure S3. Showing SIMC quantification of H3K27me3-K36me2 and H3K27me2-K36me3-containing peptides by Orbitrap MS/MS. Two synthetic peptides S1 (K27me2-K36me3) and S2 (K27me3-K36me2) of H3K27-R40, and their mixtures M1–M4 were subjected to LC-MS/MS analysis. The intensity of two types of all specific y^2+^ ions in multiple tandem mass spectra of all peptide samples from Orbitrap was summarized and showed as a SIMC profile **A–F. G** SIMC quantification of K27me3-K36me2 and K27me2-K36me3 in S1, S2, and M1–M4 samples from Orbitrap data. Constituent percentages of two isoforms of H3K27-R40me5 were indicated with different colors, K27me2-K36me3 in *red* and K27me3-K36me2 in *green*. The MS experiment was repeated three times and error bars represent the SD. (DOCX 260 kb) Additional file 4: Figure S4. Showing the intensity of two types of b^1+^, b^2+^, y^1+^, and y^2+^ specific ions in a series of tandem mass spectra of M2 by Orbitrap displayed as a chromatographic profile. Quantification was based on the peak area of two types of y11–13^2+^ ions. (DOCX 90 kb) Additional file 5: Figure S5. Showing SIMC quantification of H3K27me3-K36me2 and H3K27me2-K36me3-containing peptides digested by trypsin. Two longer synthetic peptides of H3 L20-R52me5, L1 (K27me2-K36me3) and L2 (K27me3-K36me2), were mixed with different ratios (M1–M4), and digested with trypsin for 20, 40, and 120 minutes, respectively. The results for SIMC quantification of K27me3-K36me2 and K27me2-K36me3 in M1 **A–C**, M2 **D–F**, M3 **G–I**, and M4 **J–L** samples displayed as curves. (DOCX 249 kb) Additional file 6: Figure S6. Showing **A** microscopic pictures of undifferentiated (D0) and differentiated mouse ES cells at day 2 (D2) and day 4 (D4). **B** Quantitative RT-PCR analysis of pluripotency (*Oct4* and *Nanog*) and differentiation (*Fgf5*, *T*, *Sox1* and *Gata4*) markers along ESC differentiation. Experiment was repeated three times and error bars represent the SD. (DOCX 997 kb) Additional file 7: Figure S7. Showing SIC quantification of H3K27-R40me0-6 peptides in undifferentiated (D0) and differentiated mESCs (D2 and D4). Through SIC of the precursors at MS level, the percentages of H3K27-R40me0-6 were calculated in Arg-C **A** and trypsin **B** digested samples, respectively. (DOCX 72 kb) Additional file 8: Figure S8. Showing SIMC quantification of two isoforms of H3K27-R40me5 in mESCs and differentiated cells for three repeated experiments. The isoforms were labeled with different colors, and displayed as curves, K27me2-K36me3 in *red* and K27me3-K36me2 in *green*, respectively. (DOCX 189 kb) Additional file 9: Figure S9. Showing western blot analysis of H3K27me3 levels in undifferentiated (D0) and differentiated ESCs (D2 and D4). H3 was used as the loading control. (DOCX 116 kb) Additional file 10: Figure S10. Showing SIMC quantification of S1 (K27me2-K36me3) and S2 (K27me3-K36me2) in a series of diluted solutions. A mixture containing S1 and S2 (50 wt%:50 wt%, 25 ng/μl) was diluted 10, 50, 100, and 500 times (10×, 50×, 100×, and 500×) by ddH2O, respectively. Constituent percentages of K27me2-K36me3 (*red*) and K27me3-K36me2 (*green*) were detected. The MS experiment was repeated three times and error bars represent the SD. (DOCX 79 kb)

## Abbreviations

ChIP-Seq: Chromatin immunoprecipitation sequencing
CID: Collision-induced dissociation
ddH_2_O: Double-distilled water
ESC: Embryonic stem cell
FWHM: Full width at half maximum
LC: Liquid chromatography
LIF: Leukemia inhibitory factor
MALDI: Matrix-assisted laser desorption ionization
MEF: Mouse embryonic fibroblast
mESC: Mouse embryonic stem cell
MS: Mass spectrometry
MS/MS: Tandem mass spectrometry
*m/*z: Mass-to-charge ratio
PRC2: Polycomb repressive complex 2
RP-HPLC: Reversed phased-high performance liquid chromatography
SD: Standard deviation
SIC: Selected ion chromatogram
SIMC: Specific ions of isobaric modification chromatogram
TFA: Trifluoroacetic acid

## References

[1] Li B, Carey M, Workman JL (2007). The role of chromatin during transcription. Cell..

[2] Martin C, Zhang Y (2005). The diverse functions of histone lysine methylation. Nat Rev Mol Cell Biol..

[3] Nottke A, Colaiacovo MP, Shi Y (2009). Developmental roles of the histone lysine demethylases. Development..

[4] Bannister AJ, Kouzarides T (2005). Reversing histone methylation. Nature..

[5] Bannister AJ, Kouzarides T (2004). Histone methylation: recognizing the methyl mark. Methods Enzymol..

[6] Bedford MT, Richard S (2005). Arginine methylation an emerging regulator of protein function. Mol Cell..

[7] Margueron R, Trojer P, Reinberg D (2005). The key to development: interpreting the histone code?. Curr Opin Genet Dev..

[8] Mikkelsen TS, Ku M, Jaffe DB, Issac B, Lieberman E, Giannoukos G (2007). Genome-wide maps of chromatin state in pluripotent and lineage-committed cells. Nature..

[9] Young MD, Willson TA, Wakefield MJ, Trounson E, Hilton DJ, Blewitt ME (2011). ChIP-seq analysis reveals distinct H3K27me3 profiles that correlate with transcriptional activity. Nucleic Acids Res..

[10] Cai L, Rothbart SB, Lu R, Xu B, Chen WY, Tripathy A (2013). An H3K36 methylation-engaging Tudor motif of polycomb-like proteins mediates PRC2 complex targeting. Mol Cell..

[11] Voigt P, LeRoy G, Drury WJ, Zee BM, Son J, Beck DB (2012). A symmetrically modified nucleosomes. Cell..

[12] Yuan W, Xu M, Huang C, Liu N, Chen S, Zhu B (2011). H3K36 methylation antagonizes PRC2-mediated H3K27 methylation. J Biol Chem..

[13] Aebersold R, Mann M (2003). Mass spectrometry-based proteomics. Nature..

[14] Garcia BA, Mollah S, Ueberheide BM, Busby SA, Muratore TL, Shabanowitz J (2007). Chemical derivatization of histones for facilitated analysis by mass spectrometry. Nat Protoc..

[15] Jung HR, Pasini D, Helin K, Jensen ON (2010). Quantitative mass spectrometry of histones H3.2 and H3.3 in Suz12-deficient mouse embryonic stem cells reveals distinct, dynamic post-translational modifications at Lys-27 and Lys-36. Mol Cell Proteomics.

[16] Peters AH, Kubicek S, Mechtler K, O’Sullivan RJ, Derijck AA, Perez-Burgos L (2003). Partitioning and plasticity of repressive histone methylation states in mammalian chromatin. Mol Cell..

[17] Morales Torres C, Laugesen A, Helin K (2013). Utx is required for proper induction of ectoderm and mesoderm during differentiation of embryonic stem cells. PLoS One..

[18] Shen X, Kim W, Fujiwara Y, Simon MD, Liu Y, Mysliwiec MR (2009). Jumonji modulates polycomb activity and self-renewal versus differentiation of stem cells. Cell..

[19] Shechter D, Dormann HL, Allis CD, Hake SB (2007). Extraction, purification and analysis of histones. Nat Protoc..

[20] Eng JK, McCormack AL, Yates JR (1994). An approach to correlate tandem mass spectral data of peptides with amino acid sequences in a protein database. J Am Soc Mass Spectrom..

[21] Zhang K (2007). Qualitative and quantitative analysis of lysine acetylaton and methylation in yeast histone H3. Int J Mass Spectrom..

[22] Yang L, Tu S, Ren C, Bulloch EM, Liao CL, Tsai MD (2010). Unambiguous determination of isobaric histone modifications by reversed-phase retention time and high-mass accuracy. Anal Biochem..

[23] Steen H, Mann M (2004). The ABC’s (and XYZ’s) of peptide sequencing. Nat Rev Mol Cell Biol..

[24] Perkins DN, Pappin DJ, Creasy DM, Cottrell JS (1999). Probability-based protein identification by searching sequence databases using mass spectrometry data. Electrophoresis..

[25] Bleiholder C, Suhai S, Harrison AG, Paizs B (2011). Towards understanding the tandem mass spectra of protonated oligopeptides. 2: The proline effect in collision-induced dissociation of protonated Ala-Ala-Xxx-Pro-Ala (Xxx = Ala, Ser, Leu, Val, Phe, and Trp). J Am Soc Mass Spectrom.

[26] Schwartz BL, Bursey MM (1992). Some proline substituent effects in the tandem mass spectrum of protonated pentaalanine. Biol Mass Spectrom..

[27] Olsen JV, Ong SE, Mann M (2004). Trypsin cleaves exclusively C-terminal to arginine and lysine residues. Mol Cell Proteomics..

[28] Burlingame AL, Zhang X, Chalkley RJ (2005). Mass spectrometric analysis of histone posttranslational modifications. Methods..

[29] Zhang K, Tang H, Huang L, Blankenship JW, Jones PR, Xiang F (2002). Identification of acetylation and methylation sites of histone H3 from chicken erythrocytes by high-accuracy matrix-assisted laser desorption ionization-time-of-flight, matrix-assisted laser desorption ionization-postsource decay, and nanoelectrospray ionization tandem mass spectrometry. Anal Biochem..

[30] Ren C, Zhang L, Freitas MA, Ghoshal K, Parthun MR, Jacob ST (2005). Peptide mass mapping of acetylated isoforms of histone H4 from mouse lymphosarcoma cells treated with histone deacetylase (HDACs) inhibitors. J Am Soc Mass Spectrom..

[31] Bonenfant D, Towbin H, Coulot M, Schindler P, Mueller DR, van Oostrum J (2007). Analysis of dynamic changes in post-translational modifications of human histones during cell cycle by mass spectrometry. Mol Cell Proteomics..

[32] Lee ER, Murdoch FE, Fritsch MK (2007). High histone acetylation and decreased polycomb repressive complex 2 member levels regulate gene specific transcriptional changes during early embryonic stem cell differentiation induced by retinoic acid. Stem Cells..

[33] Pasini D, Bracken AP, Agger K, Christensen J, Hansen K, Cloos PA (2008). Regulation of stem cell differentiation by histone methyltransferases and demethylases. Cold Spring Harb Symp Quant Biol..

[34] Trelle MB, Jensen ON (2007). Functional proteomics in histone research and epigenetics. Expert Rev Proteomics..

[35] Garcia BA (2009). Mass spectrometric analysis of histone variants and post-translational modifications. Front Biosci (Schol Ed)..

[36] Strahl BD, Allis CD (2000). The language of covalent histone modifications. Nature..

[37] Zheng Y, Sweet SM, Popovic R, Martinez-Garcia E, Tipton JD, Thomas PM (2012). Total kinetic analysis reveals how combinatorial methylation patterns are established on lysines 27 and 36 of histone H3. Proc Natl Acad Sci U S A..

