# Supplementary figures and images for: Cis-existence of H3K27me3 and H3K36me2 in mouse embryonic stem cells revealed by specific ions of isobaric modification chromatogram
Source: Stem Cell Res Ther. 2015 Jul 21;6(1):132. doi: 10.1186/s13287-015-0131-0 (PMC4533945; doi:10.1186/s13287-015-0131-0)

**
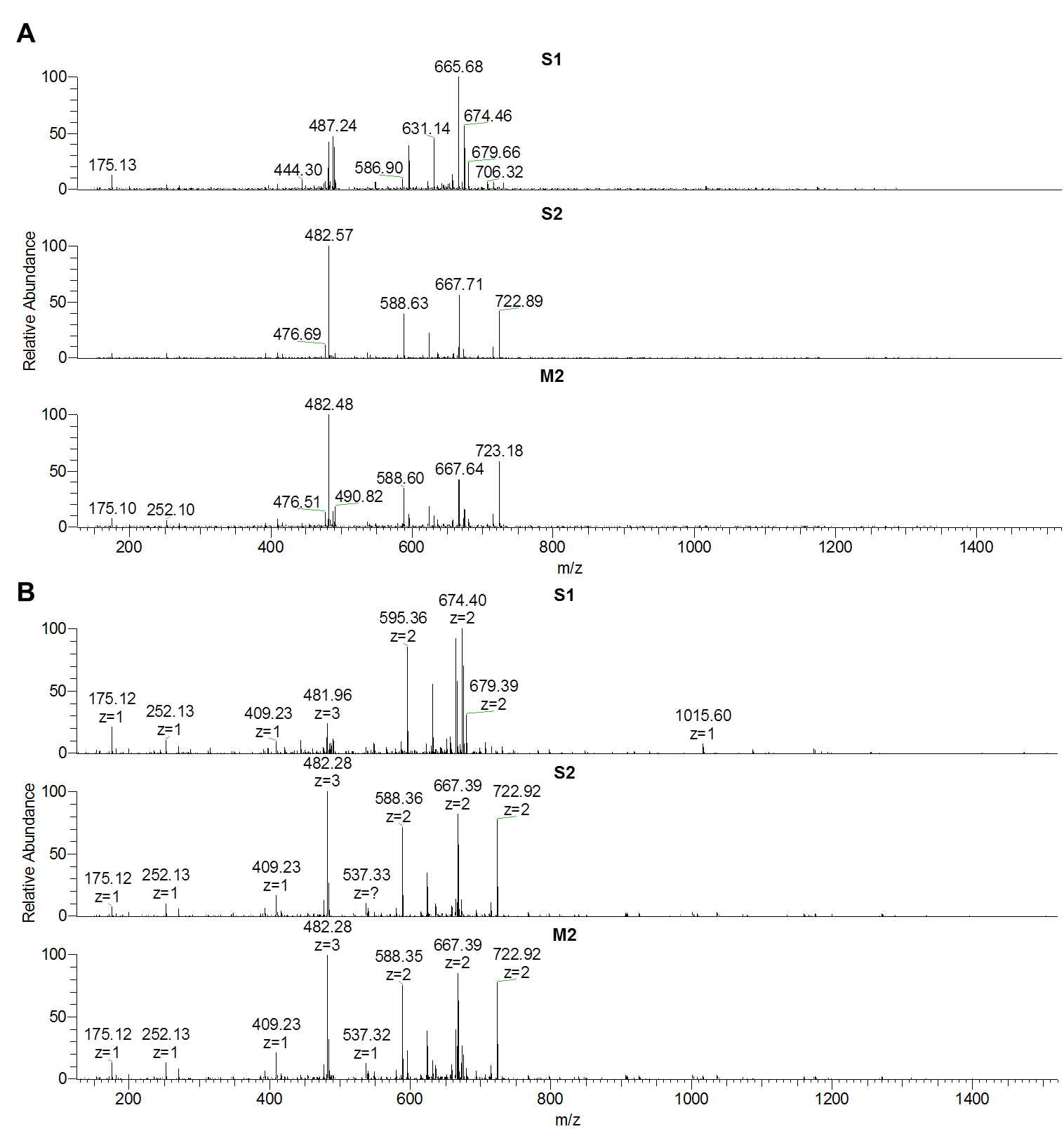
**

Supplement: Additional file 1: Figure S1. — Showing the strongest tandem mass spectra of S1 (H3K27me2-K36me3), S2 (H3K27me3-K36me2), and M2 (containing an equal amount of S1 and S2 peptides) by LTQ A and by Orbitrap B. (DOCX 123 kb) [file 13287_2015_131_MOESM1_ESM.docx]

**
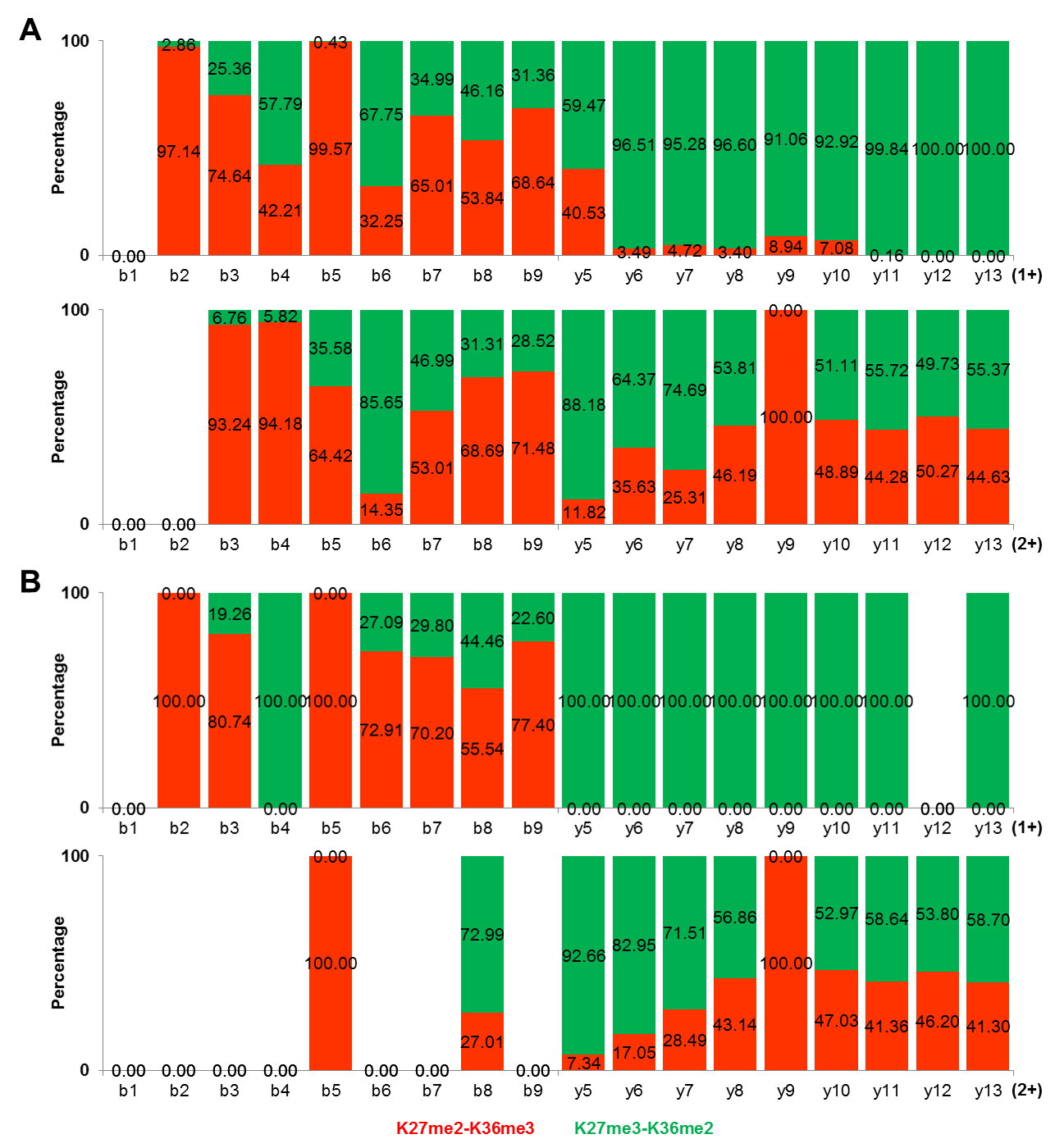
**

Supplement: Additional file 2: Figure S2. — Showing quantification of H3K27me3-K36me2 and H3K27me2-K36me3-containing peptides in M2 based on the peak area of individual specific ion by LTQ A and by Orbitrap B. All 1+ and 2+ charged b1–9 and y5–13 specific ions were examined in multiple tandem mass spectrum of M2, a mixture containing an equal amount of the two peptides of H3K27-R40me5, S1 (K27me2-K36me3) and S2 (K27me3-K36me2). The peak area of the intensity curve in Fig. 1d and Additional file 4 was designated as the cumulative intensity of each ion to calculate the ratio of each ion pair. (DOCX 204 kb) [file 13287_2015_131_MOESM2_ESM.docx]

**
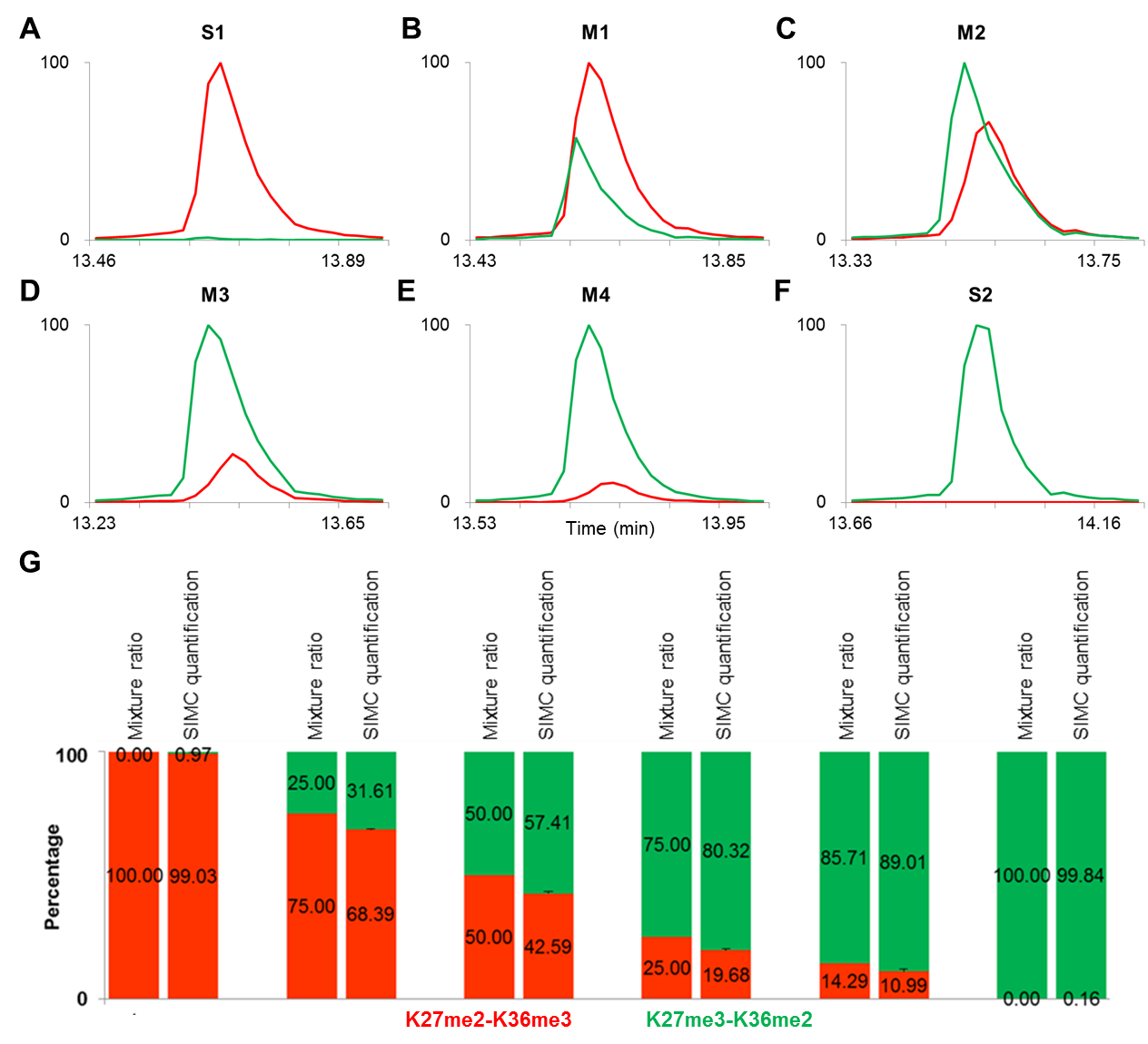
**

Supplement: Additional file 3: Figure S3. — Showing SIMC quantification of H3K27me3-K36me2 and H3K27me2-K36me3-containing peptides by Orbitrap MS/MS. Two synthetic peptides S1 (K27me2-K36me3) and S2 (K27me3-K36me2) of H3K27-R40, and their mixtures M1–M4 were subjected to LC-MS/MS analysis. The intensity of two types of all specific y2+ ions in multiple tandem mass spectra of all peptide samples from Orbitrap was summarized and showed as a SIMC profile A–F. G SIMC quantification of K27me3-K36me2 and K27me2-K36me3 in S1, S2, and M1–M4 samples from Orbitrap data. Constituent percentages of two isoforms of H3K27-R40me5 were indicated with different colors, K27me2-K36me3 in red and K27me3-K36me2 in green. The MS experiment was repeated three times and error bars represent the SD. (DOCX 260 kb) [file 13287_2015_131_MOESM3_ESM.docx]

**
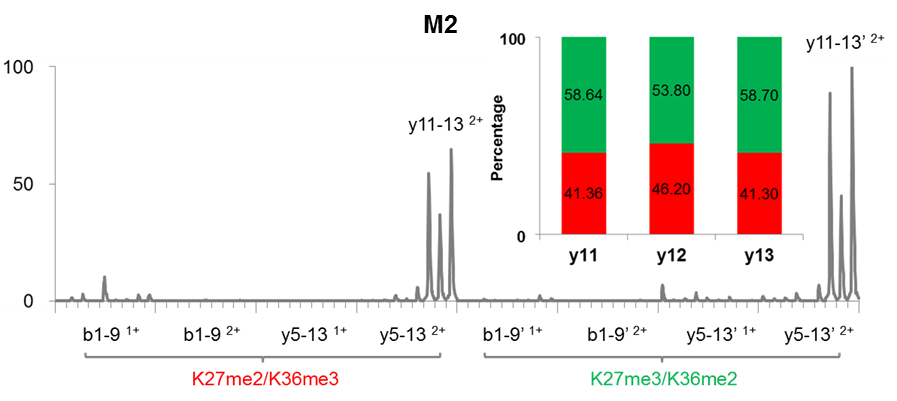
**

Supplement: Additional file 4: Figure S4. — Showing the intensity of two types of b1+, b2+, y1+, and y2+ specific ions in a series of tandem mass spectra of M2 by Orbitrap displayed as a chromatographic profile. Quantification was based on the peak area of two types of y11–132+ ions. (DOCX 90 kb) [file 13287_2015_131_MOESM4_ESM.docx]

**
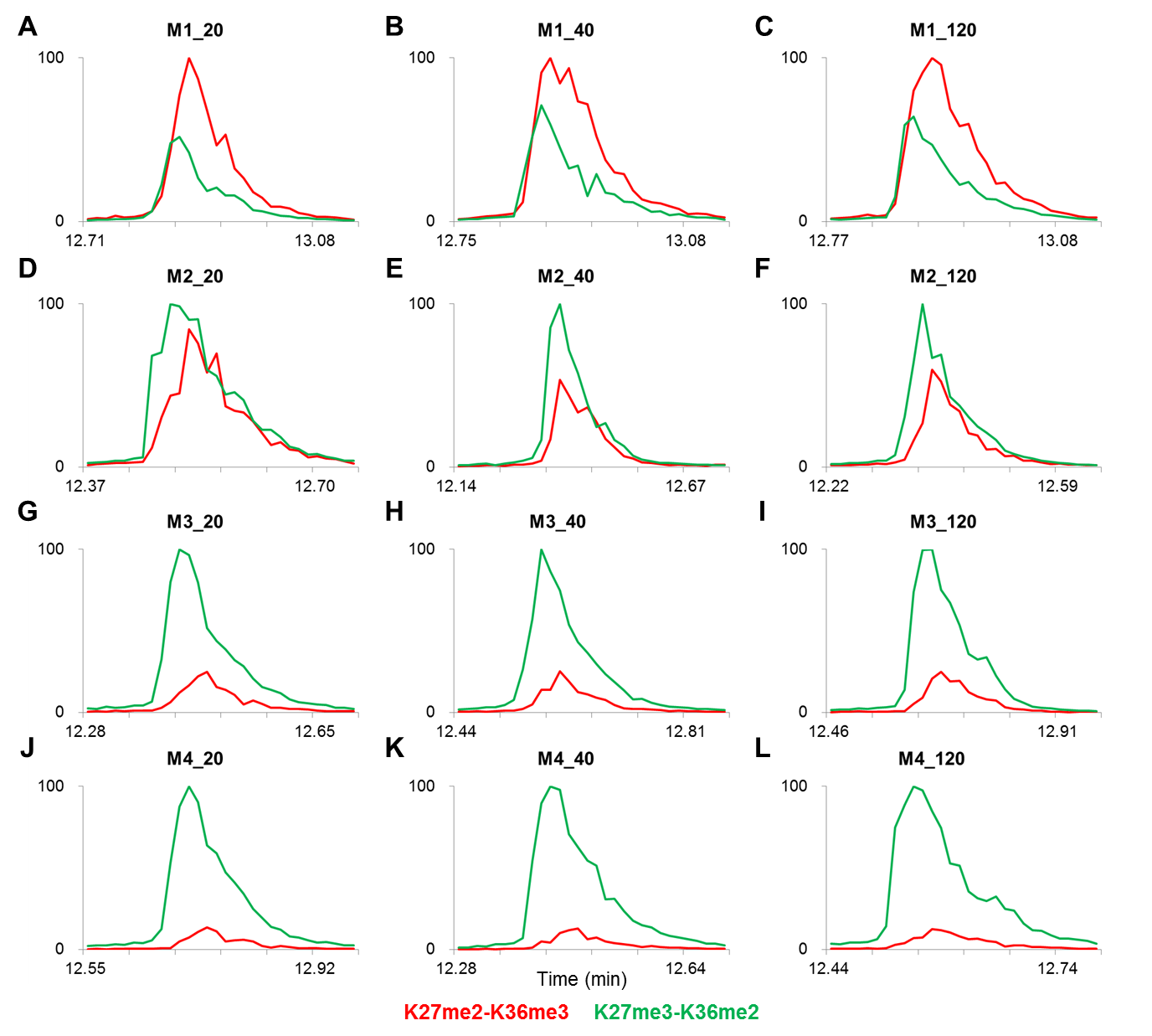
**

Supplement: Additional file 5: Figure S5. — Showing SIMC quantification of H3K27me3-K36me2 and H3K27me2-K36me3-containing peptides digested by trypsin. Two longer synthetic peptides of H3 L20-R52me5, L1 (K27me2-K36me3) and L2 (K27me3-K36me2), were mixed with different ratios (M1–M4), and digested with trypsin for 20, 40, and 120 minutes, respectively. The results for SIMC quantification of K27me3-K36me2 and K27me2-K36me3 in M1 A–C, M2 D–F, M3 G–I, and M4 J–L samples displayed as curves. (DOCX 249 kb) [file 13287_2015_131_MOESM5_ESM.docx]

**
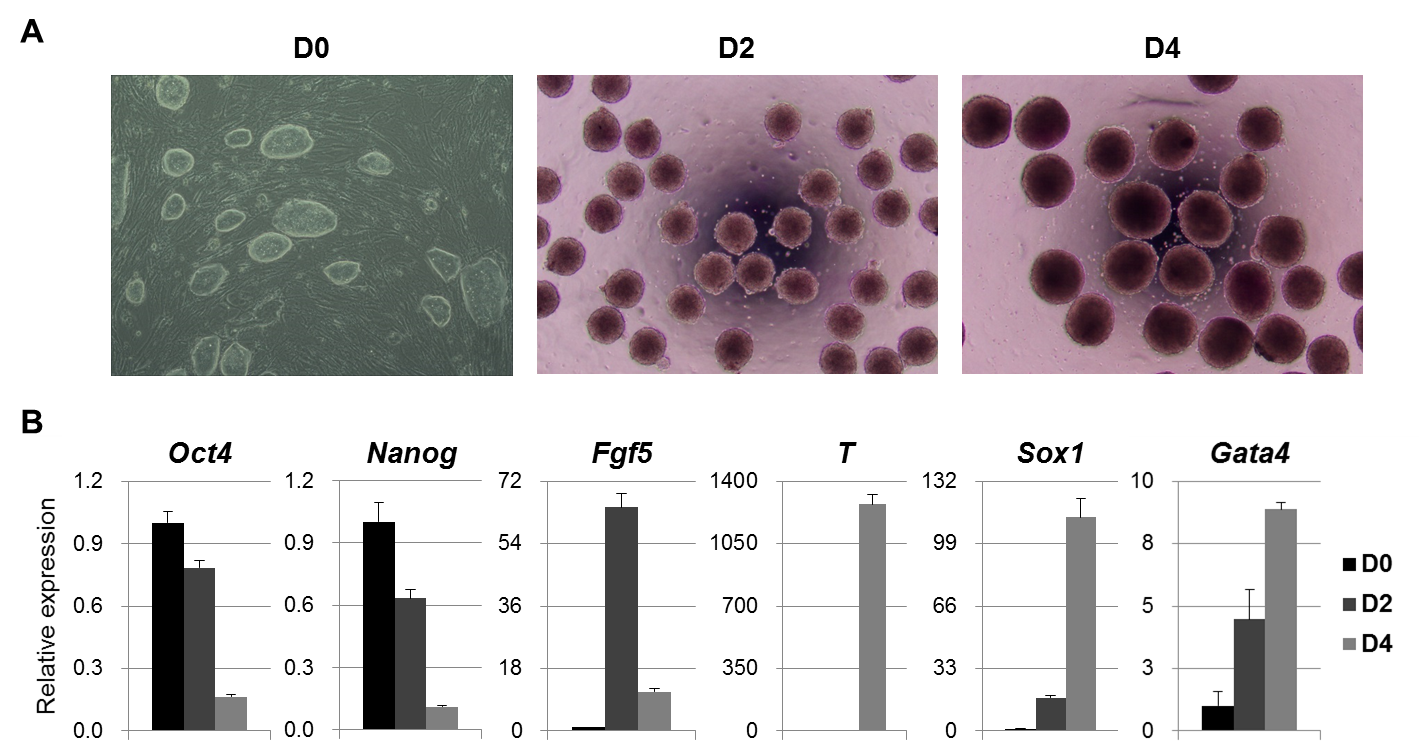
**

Supplement: Additional file 6: Figure S6. — Showing A microscopic pictures of undifferentiated (D0) and differentiated mouse ES cells at day 2 (D2) and day 4 (D4). B Quantitative RT-PCR analysis of pluripotency (Oct4 and Nanog) and differentiation (Fgf5, T, Sox1 and Gata4) markers along ESC differentiation. Experiment was repeated three times and error bars represent the SD. (DOCX 997 kb) [file 13287_2015_131_MOESM6_ESM.docx]

**
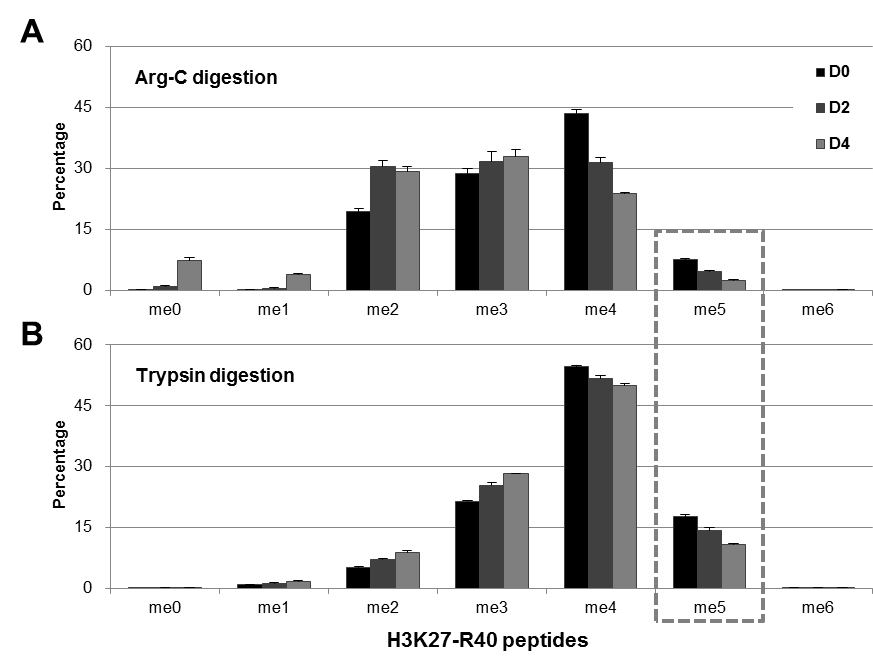
**

Supplement: Additional file 7: Figure S7. — Showing SIC quantification of H3K27-R40me0-6 peptides in undifferentiated (D0) and differentiated mESCs (D2 and D4). Through SIC of the precursors at MS level, the percentages of H3K27-R40me0-6 were calculated in Arg-C A and trypsin B digested samples, respectively. (DOCX 72 kb) [file 13287_2015_131_MOESM7_ESM.docx]

**
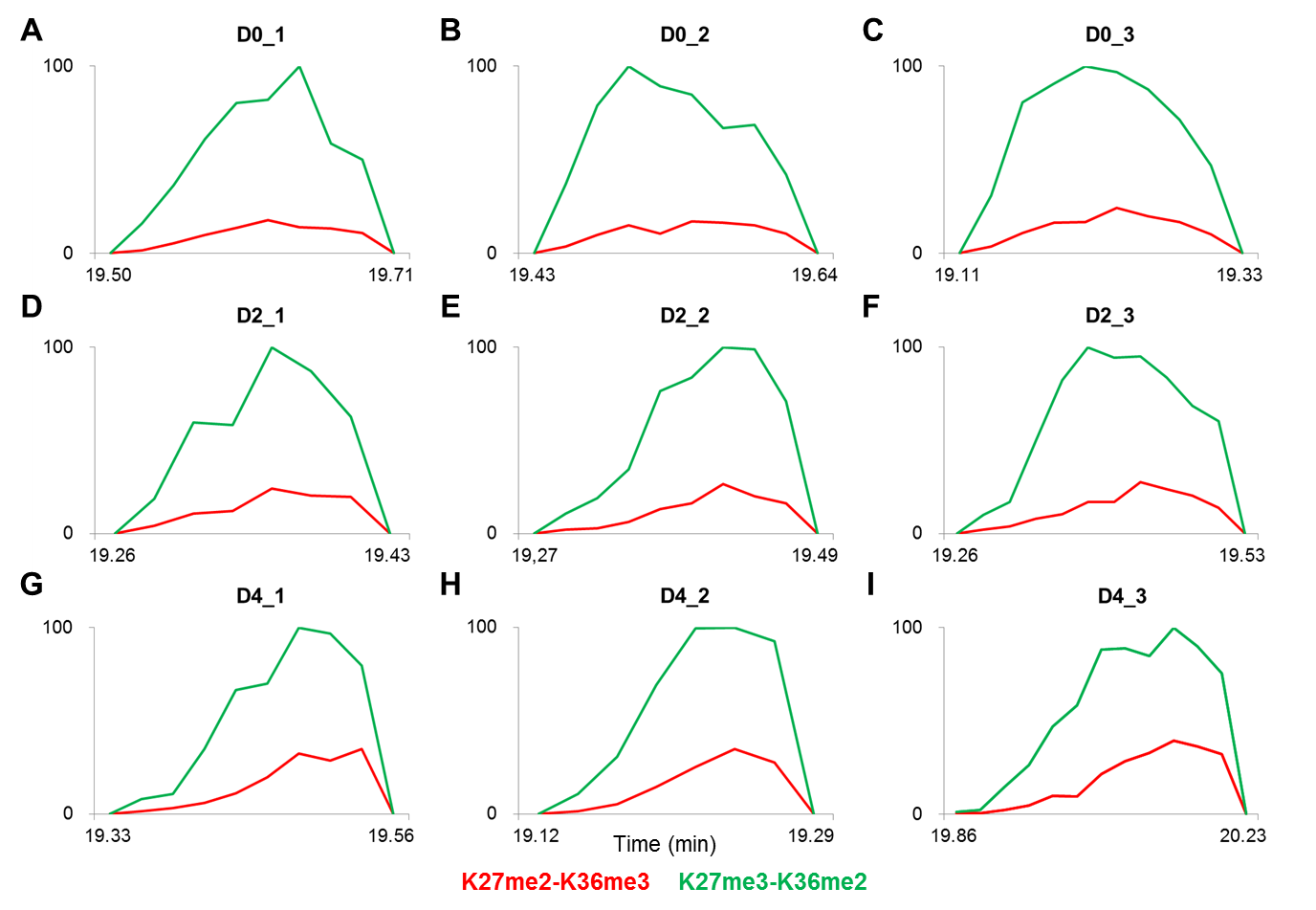
**

Supplement: Additional file 8: Figure S8. — Showing SIMC quantification of two isoforms of H3K27-R40me5 in mESCs and differentiated cells for three repeated experiments. The isoforms were labeled with different colors, and displayed as curves, K27me2-K36me3 in red and K27me3-K36me2 in green, respectively. (DOCX 189 kb) [file 13287_2015_131_MOESM8_ESM.docx]

**
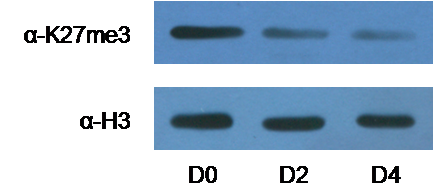
**

Supplement: Additional file 9: Figure S9. — Showing western blot analysis of H3K27me3 levels in undifferentiated (D0) and differentiated ESCs (D2 and D4). H3 was used as the loading control. (DOCX 116 kb) [file 13287_2015_131_MOESM9_ESM.docx]

**
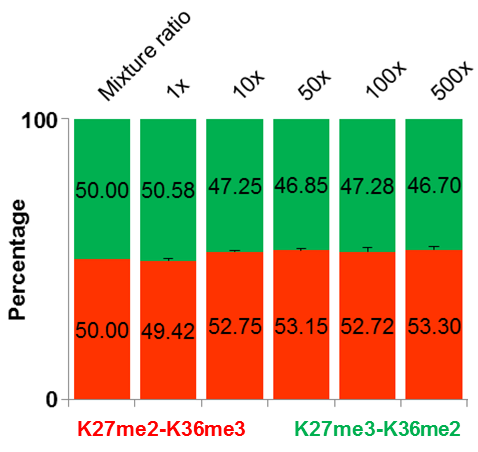
**

Supplement: Additional file 10: Figure S10. — Showing SIMC quantification of S1 (K27me2-K36me3) and S2 (K27me3-K36me2) in a series of diluted solutions. A mixture containing S1 and S2 (50 wt%:50 wt%, 25 ng/μl) was diluted 10, 50, 100, and 500 times (10×, 50×, 100×, and 500×) by ddH2O, respectively. Constituent percentages of K27me2-K36me3 (red) and K27me3-K36me2 (green) were detected. The MS experiment was repeated three times and error bars represent the SD. (DOCX 79 kb) [file 13287_2015_131_MOESM10_ESM.docx]
